# The role of serum gastric peptide ghrelin hormone level in irritable bowel syndrome at Zagazig University Hospitals

**DOI:** 10.25122/jml-2022-0089

**Published:** 2022-12

**Authors:** Sameh Mahmoud Abdel Monem, Elsaid Galal Elbadrawy, Sally Mahmoud Shalaby, Doaa Mahmoud Hendawy, Sherif Mahmoud Hassan, Nagla Abd Almonem

**Affiliations:** 1 Tropical Medicine Department, Faculty of Medicine, Zagazig University, Zagazig, Egypt; 2 Medical Biochemistry & Molecular Biology Department, Faculty of Medicine, Zagazig University, Zagazig, Egypt

**Keywords:** irritable bowel syndrome, ghrelin, diarrhea, constipation, ESR – erythrocyte sedimentation rate, FGID – functional gastrointestinal disorder, GH – growth hormone, BMI – body mass index, IBS – Irritable bowel syndrome, IBS-C – Irritable bowel syndrome constipation, IBS-D – Irritable bowel syndrome diarrhea, IBS-M – Irritable bowel syndrome mixed diarrhea and constipation, IBS-U – Irritable bowel syndrome unclassified, TSH – thyroid-stimulating hormone

## Abstract

Generalized dysmotility of the gastrointestinal tract develops in individuals with irritable bowel syndrome (IBS). The ghrelin hormone appears to be critical in controlling gastrointestinal motility. We aimed to evaluate serum ghrelin levels in people with IBS and to demonstrate its role in IBS pathophysiology. This study included 32 individuals with IBS (16 with constipation and 16 with diarrhea) and 16 healthy individuals as controls. Blood specimens were collected from patients and controls following an overnight fast. Total ghrelin level was detected in plasma by commercially available ELISA Kit. There were significant differences in the serum levels of ghrelin between the control group and both types of IBS. The mean±SD of ghrelin level in the control group was 2.608±0.714 pg/ml, and that of both types of IBS was 5.782±2.450 pg/ml (P-value<0.001). There was a significant variation between the control and IBS-D groups (mean±SD: 7.838±1.687 pg/ml, p-value<0.001). Also, we indicated a considerable difference between the control and IBS-C groups (mean±SD: 3.726±0.740 pg/ml, P-value<0.001). In comparing the IBS-D group and IBS-C group, we found a highly considerable variation between the two groups (p-value<0.001). This means that serum ghrelin levels were significantly greater in IBS-D than in IBS-C and the control group. Our findings concluded that serum ghrelin level was higher among the IBS-D group than in the IBS-C and control groups. The ghrelin hormone may play a vital role in IBS pathophysiology.

## INTRODUCTION

Irritable bowel syndrome (IBS) is one of the most frequent gastrointestinal issues with uncertain pathogenesis. It is defined as an extremely prevalent debilitating and chronic functional gastrointestinal (FGID) disorder that presents clinically with diverse phenotypes with various combinations of symptoms [1]. Irritable bowel syndrome is a condition that manifests through abdominal discomfort or distress, abdominal bloating or distention, and changes in bowel habits [2]. It affects nearly 15 to 25% of the world's population, being a common diagnosis in nearly 20–40% of all gastroenterological consultations [3]. Laboratory and radiographic results and endoscopic investigations are normal in these individuals, and diagnosis is made primarily based on symptoms, which vary from mild to severe in different patients and lead to interference with daily activity [4]. IBS affects the quality of life in society, which is important from an economic point of view, affecting the ability to work and consuming excessive healthcare resources [5]. Genetic factors, past infections, mucosal inflammation, psychosocial factors, impaired motility, impaired intestinal flora, and gastrointestinal hormones play a role in IBS pathogenesis [6]. IBS is categorized into four subtypes depending on bowel habits: constipation, diarrhea, mixed IBS, and un-sub-typed IBS [7]. Ghrelin is a peptide hormone composed of 28-amino acid derived from the stomach. Ghrelin's tiny concentrations were indicated in the small intestine and hypothalamus; however, it mostly originates from the stomach from the endocrine cells associated with the oxyntic mucosa [8]. Furthermore, it has various roles, the most well-known being its secreting impact on growth hormone (GH). Additionally, ghrelin increases hunger and feeding and performs the main function in energy consumption [9]. Also, ghrelin has been demonstrated to increase motility of the stomach, large and small intestine, patients with IBS having gastrointestinal dysmotility [10]. In this study, we targeted the potential role of ghrelin-associated IBS pathophysiology.

## MATERIAL AND METHODS

This study included 32 individuals diagnosed with irritable bowel syndrome (IBS) using the Rome IV criteria (14 males and 18 females; mean age 43.53±13.38 years; range 19–65 years). We excluded: mixed types of IBS, patients receiving treatment for IBS, persons with a background of prior gastrointestinal surgery, documented malignancy, autoimmune diseases, endocrinal diseases IBD and any other cause of functional or organic GIT disease, chronic liver disorder, diabetes mellitus, or chronic kidney disorder and pregnant women. These patients had no concerning symptoms. Patients were classified into two subtypes using the Rome IV criteria, which describes IBS as minimum 3 months of periodic abdominal pain with at minimum one day of pain/week, in addition to any two or more of the preceding signs: pain correlated with defecation; ailments in the stool form/consistency; and reduced stool frequency (less than three bowel movements/week). Thus, 16 patients (11 females and 5 males; mean age 44.6 years; vary19–65) had IBS-constipation, and another 16 patients had IBS-diarrhea (9 males and 7 females; mean age 42.4 years; vary 22–64). In addition, 16 healthy volunteers (11 males and 5 females; mean age 47 years; range 25–64) without any chronic or recurrent gastrointestinal symptoms were included as controls. A 10-mL blood sample was taken from each individual after resting for at least 15 minutes between 8 and 9 a.m. following a 12–14 hour fast. Two quantities of blood were drawn: 1.5 mL of whole blood samples were taken in a fluoride-containing tube to estimate blood glucose, and 1.5 mL of blood was taken in sterile EDTA-containing tubes for CBC analysis. To determine the erythrocyte sedimentation rate (ESR), 2 mL of blood was obtained in sterile sodium citrate-containing tubes. The remaining portion (5 mL) was left at room temperature for 30 to 60 minutes to allow for spontaneous clotting before centrifuging at 4,000 rpm for 10 minutes. Serum samples were utilized to determine the lipid profile, C-reactive protein, liver and kidney function tests, serum ghrelin, and autoantibodies such as ANA, Anti-dsDNA, and P-ANCA. Serum samples were stored at a temperature of -20°C.

### Ghrelin detection

Blood specimens were taken in pre-chilled polypropylene tubes comprising EDTA (1 mg/ml blood) and promptly centrifuged at 4°C for 10 minutes at 3000 rpm. Plasma was obtained and kept at -20°C until analysis. Total serum ghrelin concentrations were determined by the available commercially Human Ghrelin ELISA kit (Competitive ELISA) 96 Tests (My Bio Source Catalog Number: MBS720885), (California, USA). This study was conducted between October 2019 and October 2020 in the Departments of Tropical Medicine and Medical Biochemistry and Molecular Biology, Faculty of Medicine, Zagazig University Hospitals. The study was authorized by Zagazig University's institutional review board (IRB). All subjects signed informed consent. Blood specimens were gathered from patients and controls following an overnight fast.

### Statistical analysis

All data were collected and analyzed statistically utilizing SPSS 22 for Windows (IBM Corp., Armonk, New York, USA) and MedCalc 13 for Windows (IBM Corp., Armonk, New York, USA) (MedCalc Software bvba, Ostend, Belgium). Continuous quantitative variables were represented as mean, standard deviation, and median, while categorical qualitative variables were represented as absolute and relative frequencies (number and percentage). Samples were taken independently to evaluate two sets of regularly distributed data. Student's t-test was employed, and the Mann-Whitney U test was utilized for non-normally distributed data. A P-value less than 0.05 was regarded statistically significant, less than 0.001 was considered highly significant, and higher than or equal to 0.05 was considered insignificant. In addition, Spearman's rank correlation coefficient was performed to determine correlations between ghrelin and study parameters.

## RESULTS

### Serum ghrelin detection

There was a highly significant statistical variation in serum ghrelin concentrations between control and IBS groups (Table 1), with IBS patients having higher levels of ghrelin.

**Table 1:** The levels of ghrelin concentration in the control and IBS groups.

	Control (n=16)	IBS cases (n=32)	Test	p-value (Sig.)
**Serum Ghrelin (pg/ml)**				
Mean±SD	2.608±0.714	5.782±2.450	-4.833 ^c^	<0.001 (HS)
Median	2.800	4.720		
Range	1.310–4.010	2.290–11.032		

c – Mann Whitney U test; P-values less than 0.05 are significant; Sig – Significance.

Furthermore, patients with IBS-D and IBS-C had significantly higher levels of ghrelin serum concentrations compared to the control group (p<0.001) (Tables 2 and 3).

**Table 2:** The levels of ghrelin concentration in the control and IBS-D groups.

	Control (n=16)	IBS-D cases (n=16)	Test	p-value (Sig.)
**Serum Ghrelin (pg/ml)**				
Mean±SD	2.608±0.714	7.838±1.687	-11.418	<0.001 (HS)
Median	2.800	7.865		
Range	1.310–4.010	4.760–11.032		

b – Independent samples Student's t-test; ^c^ – Mann Whitney U test; P-values less than 0.05 are significant; Sig – Significance.

**Table 3:** The levels of ghrelin concentration in the control and IBS-C groups.

	Control (n=16)	IBS-C cases (n=16)	Test	p-value (Sig.)
**Serum Ghrelin (pg/ml)**				
Mean±SD	2.608±0.714	3.726±0.740	-4.350 ^b^	<0.001 (HS)
Median	2.800	4.015		
Range	1.310–4.010	2.290–4.680		

b – Independent samples Student's t-test; ^c^ – Mann Whitney U test; P-values less than 0.05 are significant; Sig – Significance.

There were also significant differences in the ghrelin serum concentrations between IBS-D and IBS-C cases, with greater levels in IBS-D patients (Table 4).

**Table 4:** The levels of ghrelin concentration in IBS-D and IBS-C cases.

	IBS-D (n=16)	IBS-C cases (n=16)	Test	p-value (Sig.)
**Serum Ghrelin (pg/ml)**				
Mean±SD	7.838±1.687	3.726±0.740	8.924 ^b^	<0.001 (HS)
Median	7.865	4.015		
Range	4.760–11.032	2.290–4.680		

b – Independent samples Student's t-test; ^c^ – Mann Whitney U test; P-values less than 0.05 are significant; Sig – Significance.

## DISCUSSION

IBS is defined by periodic abdominal discomfort lacking anatomical or biochemical anomalies. The Rome IV criteria set the bar for diagnosing IBS [11]. In humans, ghrelin is generated using P/D1 cells present throughout the stomach mucosa and is primarily produced by this distinct sort of stomach cells. Ghrelin's blood level is determined by its secretion, breakdown, and clearance rates [12]. Ghrelin levels in the plasma increase with fasting and decrease during eating [13]. Because ghrelin increases gastrointestinal motility, this might explain intermittent diarrhea and constipation observed in these patients [2]. This work aimed to investigate the relationship between serum ghrelin and irritable bowel syndrome by measuring the serum ghrelin level in individuals with irritable bowel syndrome and examining its role in IBS pathogenesis. According to basic data in our study, there was no significant difference regarding sex, age, body mass index, residence, marital status, and special habits between the control group and the other two groups (Tables 5, 6). In contrast, Drossman et al. found higher rates of IBS in women [14], while the results of Lovell & Ford were the same as our results regarding gender [15]. Also, we found no significant difference between the control and IBS-D groups. In contrast to our result, AlAmeel et al. identified more males recruited in the diarrhea-predominant group [16]. However, Şahin-Eryılmaz et al. found that there were significantly more female participants in IBS-C than in the other groups, whereas there was no variation between IBS-D and the control group [5]. On the other hand, we found a significant variation in gender between the control group and IBS-C groups. According to stool analysis in our study, there was no significant variation between the control group and the other two groups (IBS-D and IBS-C). There was a highly significant difference in Rome IV criteria between control and IBS cases regarding abdominal pain, relation to defecation and form, and significant difference in frequency (Table 7). When comparing the control and IBS-D groups, the difference was highly significant regarding all the criteria (Table 8). The same comparison between the control and the IBS-C group was highly significant for abdominal pain, relation to defecation and form but was not significant regarding frequency (Table 9). There was no significant difference in the complete blood count in our study between the control group and both IBS groups. Additionally, there was no significant variation in the lipid profile and blood sugar between the control and IBS groups. Moreover, there was no significant difference in thyroid function tests between the control and IBS groups. Against our results, Khadka et al. showed that thyroid dysfunction was more prevalent in those associated with irritable bowel syndrome (19%) than in the overall population [17].

**Table 5:** Basic characteristics of the control and IBS groups.

Basic characteristics	Control (n=16)		IBS cases (n=32)		Test	p-value (Sig.)
	No.	%	No.	%		
**Sex**						
Male	11	68.8%	14	43.8%	2.671 ^a^	0.102 (NS)
Female	5	31.2%	18	56.2%		
**Age (years)**						
Mean±SD	47±11.61		43.53±13.38		0.883b	0.382 (NS)
Median (Range)	49.50 (25–64)		44.50 (19–65)			
**BMI (kg/m^2^)**						
Mean±SD	28.78±3.59		29.77±4.47		-0.764 ^b^	0.449 (NS)
Median (Range)	29.50 (23.20–33.60)		29.25 (21.30–39.70)			
**Residence**						
Urban	9	56.2%	18	56.2%	0.000 ^a^	1.000 (NS)
Rural	7	43.8%	14	43.8%		
**Marital status**						
Married	14	87.5%	26	81.2%	0.300 ^a^	0.701 (NS)
Unmarried	2	12.5%	6	18.8%		
**Special habits**						
No	10	62.5%	24	75%	0.807 ^a^	0.503 (NS)
Yes (smoking)	6	37.5%	8	25%		

a – Chi-square test; ^b^ – Independent samples Student's t-test; p-value<0.05 is significant; Sig – Significance.

**Table 6:** Basic characteristics of the control and IBS-C cases.

Basic characteristics	Control (n=16)		IBS-C cases (n=16)		Test^a^	p-value (Sig.)
	No.	%	No.	%		
**Sex**						
Male	11	68.8%	5	31.2%	4.500	0.034 (S)
Female	5	31.2%	11	68.8%		

a – Chi-square test; p-value<0.05 is significant; Sig – Significance.

**Table 7:** Rome IV criteria in the control and IBS cases.

Rome IV criteria	Control (n=16)		IBS cases (n=32)		Test^a^	p-value (Sig.)
	No.	%	No.	%		
**Stool analysis**						
Normal	11	68.8%	21	65.6%	0.047	0.829 (NS)
Undigested	5	31.2%	11	34.4%		
**Abdominal pain**						
No pain	16	100%	0	0%	48.000	<0.001 (HS)
Pain	0	0%	32	100%		
**Relation to defecation**						
Not related	16	100%	0	0%	48.000	<0.001 (HS)
No relief	0	0%	10	31.2%		
Relief by defecation	0	0%	22	68.8%		
**Frequency**						
Every three days	0	0%	2	6.2%	19.689	0.012 (S)
Every two days	0	0%	1	3.1%		
Every other day	2	12.5%	4	12.5%	19.689	0.012 (S)
Once/day	12	75%	5	15.6%		
Twice/day	2	12.5%	5	15.6%		
Three times/day	0	0%	2	6.2%		
Four-time/day	0	0%	5	15.6%		
Five times/day	0	0%	6	18.8%		
Six times/day	0	0%	2	6.2%		
**Form**						
Normal	16	100%	0	0%	48.000	<0.001 (HS)
Separated hard lumps	0	0%	7	21.9%		
Sausage shaped but lumpy	0	0%	4	12.5%		
Sausage shaped with cracks	0	0%	5	15.6%		
Sausage like snake and soft	0	0%	3	9.4%		
Fluffy with ragged edges	0	0%	8	25%		
Watery	0	0%	5	15.6%		

a – Chi-square test; p-value<0.05 is significant; Sig. – Significance.

**Table 8:** Rome IV criteria in the control and IBS-D cases.

Rome IV criteria	Control (n=16)		IBS-D cases (n=16)		Test^a^	p-value (Sig.)
	No.	%	No.	%		
**Stool analysis**						
Normal	11	68.8%	10	62.5%	0.139	0.710 (NS)
Undigested	5	31.2%	6	37.5%		
**Abdominal pain**						
No pain	16	100%	0	0%	32.000	<0.001 (HS)
Pain	0	0%	16	100%		
**Relation to defecation**						
Not related	16	100%	0	0%	32.000	<0.001 (HS)
No relief	0	0%	7	43.8%		
Relief by defecation	0	0%	9	56.2%		
**Frequency**						
Every other day	2	12.5%	0	0%	29.333	<0.001 (HS)
Once/day	12	75%	0	0%		
Twice/day	2	12.5%	1	6.2%		
Three times/day	0	0%	2	12.5%		
Four-time/day	0	0%	5	31.2%		
Five times/day	0	0%	6	37.5%		
Six times/day	0	0%	2	12.5%		
**Form**						
Normal	16	100%	0	0%	32.000	<0.001 (HS)
Sausage like snake and soft	0	0%	3	18.8%		
Fluffy with ragged edges	0	0%	8	50%		
Watery	0	0%	5	31.2%		

a – Chi-square test; p-value<0.05 is significant; Sig. – Significance.

**Table 9:** Rome IV criteria in the control and IBS-C cases.

Rome IV criteria	Control (n=16)		IBS-C cases (n=16)		Test^a^	p-value (Sig.)
	No.	%	No.	%		
**Stool analysis**						
Normal	11	68.8%	11	68.8%	0.000	1.000 (NS)
Undigested	5	31.2%	5	31.2%		
**Abdominal pain**						
No pain	16	100%	0	0%	32.000	<0.001 (HS)
Pain	0	0%	16	100%		
**Relation to defecation**						
Not related	16	100%	0	0%	32.000	<0.001 (HS)
No relief	0	0%	3	18.8%		
Relief by defecation	0	0%	13	81.2%		
**Frequency**						
Every three days	0	0%	2	12.5%	7.216	0.125 (NS)
Every two days	0	0%	1	6.2%		
Every other day	2	12.5%	4	25%		
Once/day	12	75%	5	31.2%		
Twice/day	2	12.5%	4	25%		
**Form**						
Normal	16	100%	0	0%	32.000	<0.001 (HS)
Separated hard lumps	0	0%	7	43.8%		
Sausage shaped but lumpy	0	0%	4	25%		
Sausage shaped with cracks	0	0%	5	31.2%		

a – Chi-square test; p-value<0.05 is significant; Sig. – Significance.

In comparing acute phase reactants (CRP & ESR), there was no significant difference between the control group and both IBS groups. Our study indicated a significant variation between the control group and both types of IBS regarding serum levels of ghrelin. Furthermore, the mean±SD of ghrelin serum levels in the control group was 2.608±0.714 pg/ml and 5.782±2.450 pg/ml in both types of IBS. Furthermore, there was a high variation in the serum ghrelin levels between the control group (2.608±0.714 pg/ml), IBS-D (7.838±1.687 pg/ml), and IBS-C (3.726±0.740 pg/ml) groups. When comparing the serum levels of ghrelin between IBS-C and IBS-D, we discovered a significant difference between the two groups, with the mean±SD of IBS-D group 7.838±1.687 pg/ml and IBS-C 3.726±0.740 pg/ml. This suggests that IBS-D patients had considerably higher serum ghrelin levels than IBS-C patients or the control group. According to results obtained by El-Salhy [3] et al., the median ghrelin level in IBS-D was 3.29 (1.2–12.7), in IBS-C, 1.49 (0.82–7.08), and the control group 1.5 (0.2–3.7). Plasma ghrelin levels were considerably lower in IBS-C than in IBS-D and the control group, which is consistent with our findings. However, a comparable variation between IBS-C and the control group was not observed (p=0.156). El-Salhy et al. indicated no difference in the ghrelin plasma levels between IBS individuals and controls with higher levels of ghrelin in IBS-D followed by IBS-C and then by the control group [2, 3]. Sjölund K et al. reported that ghrelin concentrations in IBS individuals were comparable to those of the control group [18].

Furthermore, we compared the two IBS groups (IBS-D & IBS-C) and found no significant difference between the two groups regarding descriptive characteristics (age, sex, BMI, residence, marital status, and special habits). Although the comparison regarding Rome IV criteria showed a highly significant difference between the IBS-D and IBS-C regarding frequency and form, there was no significant difference regarding relation to defecation and abdominal pain, or the stool analysis between both groups. According to the complete blood picture, liver function test (protein, albumin, total & direct serum bilirubin, ALT, and AST), kidney function (blood urea nitrogen and serum creatinine), lipid profiling and blood sugar, thyroid function experiments (Free T3, TSH, Free T4), acute phase reactants (ESR & CRP), there was no significant variation between the two groups (IBS-D & IBS-C). Some of the limitations of this study are the small sample size and financial resources.

## CONCLUSION

Our findings concluded that serum ghrelin level is higher among the IBS-D group than in the IBS-C and control groups. The ghrelin hormone may play a vital role in IBS pathophysiology.

## Personal thanks

We want to thank the tropical Medicine Department, Zagazig University, Medical Biochemistry Department, Zagazig University cell culture, and molecular biology lab.
